# Identification of Potential Key Genes and Pathways in Enzalutamide-Resistant Prostate Cancer Cell Lines: A Bioinformatics Analysis with Data from the Gene Expression Omnibus (GEO) Database

**DOI:** 10.1155/2020/8341097

**Published:** 2020-07-16

**Authors:** Long Zheng, Xiaojie Dou, Xiaodong Ma, Wei Qu, Xiaoshuang Tang

**Affiliations:** ^1^Department of Nuclear Medicine, The Second Affiliated Hospital of Xi'an Jiaotong University, Shaanxi 710004, China; ^2^Department of Urology, The Second Affiliated Hospital of Xi'an Jiaotong University, Shaanxi 710004, China

## Abstract

Enzalutamide (ENZ) has been approved for the treatment of advanced prostate cancer (PCa), but some patients develop ENZ resistance initially or after long-term administration. Although a few key genes have been discovered by previous efforts, the complete mechanisms of ENZ resistance remain unsolved. To further identify more potential key genes and pathways in the development of ENZ resistance, we employed the GSE104935 dataset, including 5 ENZ-resistant (ENZ-R) and 5 ENZ-sensitive (ENZ-S) PCa cell lines, from the Gene Expression Omnibus (GEO) database. Integrated bioinformatics analyses were conducted, such as analysis of differentially expressed genes (DEGs), Gene Ontology (GO) enrichment analysis, Kyoto Encyclopedia of Genes and Genomes (KEGG) pathway enrichment analysis, protein-protein interaction (PPI) analysis, gene set enrichment analysis (GSEA), and survival analysis. From these, we identified 201 DEGs (93 upregulated and 108 downregulated) and 12 hub genes (AR, ACKR3, GPER1, CCR7, NMU, NDRG1, FKBP5, NKX3-1, GAL, LPAR3, F2RL1, and PTGFR) that are potentially associated with ENZ resistance. One upregulated pathway (hedgehog pathway) and seven downregulated pathways (pathways related to androgen response, p53, estrogen response, TNF-*α*, TGF-*β*, complement, and pancreas *β* cells) were identified as potential key pathways involved in the occurrence of ENZ resistance. Our findings may contribute to further understanding the molecular mechanisms of ENZ resistance and provide some clues for the prevention and treatment of ENZ resistance.

## 1. Introduction

Given its rapidly increasing morbidity and mortality, prostate cancer (PCa) ranks first in new cases and second in deaths among cancers [1] and has thus attracted increasing research efforts in the fields of basic science and clinical medicine. The proliferation and progression of PCa strongly rely on androgen receptor (AR) signaling [2, 3]. Therefore, androgen deprivation therapy (ADT) has emerged as the first-line choice for PCa treatment [2]. Enzalutamide (ENZ), also called MDV3100, is a second-generation ADT that can eventually impair AR translocation activity, inhibiting the transcription of drivers located downstream in the AR cascade and inhibiting the proliferation and progression of PCa [4]. In metastatic castration-resistant prostate cancer (mCRPC), ENZ has also exhibited excellent therapeutic efficacy. A phase 3 clinical trial demonstrated that administration of ENZ prolonged the survival time from 13.6 months to 18.4 months in end-stage mCRPC patients who had suffered docetaxel failure [4, 5]. However, some patients (approximately 20%~40%) observed no remarkable decrease in prostate-specific antigen (PSA) after receiving ENZ; that is, they exhibited inherent resistance to ENZ [6]. In addition, even patients who respond well still ultimately develop resistance to ENZ with long-term use [6].

The mechanisms of ENZ resistance can be roughly classified into two aspects, AR-dependent and AR-independent mechanisms [7]. The AR-dependent mechanism is based on the continuous AR activation seen after castration [7]. One reason is alterations of the AR gene, such as amplifications and mutations, which lead to either abnormally high AR expression or adverse effect on the ligand-binding site [8, 9]. That is, AR amplifications increase the sensitivity of PCa cells in a low-androgen environment, while mutations elevate the affinity and specificity of the binding domain, even inducing unwanted activation by nonandrogen modules. Another mechanism is the emergence of AR splice variants, which are a series of truncated proteins derived from the alternative cleavage of AR. AR-V7 is the most famous for its strong transcription-promoting function in the AR signal cascade with a lack of androgen binding sites [10]. Other pathways, such as PI3K/AKT/PTEN, glucocorticoid receptor (GR), NF-*κ*B/p52, and Wnt/*β*-catenin, are also involved in ENZ resistance [11–14]. Some of them result in drug tolerance via crosstalk or reciprocal feedback with AR signaling, while others stimulate cell proliferation and cancer progression in their own manner.

With the utilization of next-generation RNA sequencing (RNA-seq), large-scale data on significant genes and pathways related to cancer evolution have been generated. In the field of PCa ENZ resistance, a few studies have been conducted, and diverse signatures related to pathways such as Wnt signaling [15] and CREB5 [16] have been found. However, there is a paucity of efforts using systematic and integrated bioinformatics methods to uncover key genes and pathways of ENZ resistance at the genomic level. It is reasonable to believe that other crucial molecules still exist and need to be elucidated.

In this study, we performed integrated bioinformatics analysis based on data from ENZ-resistant (ENZ-R) and ENZ-sensitive (ENZ-S) cells that were acquired from the Gene Expression Omnibus (GEO) database to identify potential key genes and pathways for ENZ resistance in PCa at the genome-wide level.

## 2. Materials and Methods

### 2.1. Dataset Searching

Relevant genome-level microarray data were retrieved from the GEO database by using a combination of specific index words, such as “prostate cancer,” “prostate adenocarcinoma,” “enzalutamide,” “MDV3100,” “resistance,” and “drug resistance.” Three gene sets (GSE104935, GSE64143, and GSE120005) that contain genomic expression data of ENZ-R and ENZ-S PCa cells were further considered. Since two of the gene sets (GSE64143 and GSE120005) provided expression data for nonduplicate samples that were unfit for further bioinformatics analysis, GSE104935, which contains samples of ENZ-S and ENZ-R LNCaP cells, was ultimately screened for our current analysis.

### 2.2. Genomic Analysis of Differentially Expressed Genes (DEGs)

The original expression matrix of GSE104935 was acquired via the GEO website. Subsequently, raw data were arranged as files of expression matrix and sample type by use of both Microsoft Excel 2016 and Perl. Normalization of expression matrix data was executed by the R command *normalizeBetweenArrays*, while the DEG analysis was performed by the limma R package. The two cutoff values for the DEGs were an expression fold change (FC) > 2 and a *P* value < 0.05. Volcano and heat map plots were generated to show the distribution and expression of identified DEGs.

### 2.3. Gene Ontology (GO) and Kyoto Encyclopedia of Genes and Genomes (KEGG) Pathway Enrichment Analyses

With the aim of investigating the functional enrichment of DEGs, GO and KEGG term enrichment analyses were conducted on the Database for Annotation, Visualization and Integrated Discovery (DAVID) website (https://david.ncifcrf.gov) [17]. DEG symbols were uploaded to the DAVID website, and the outcomes of GO and KEGG analyses were given automatically by the web tools. The analysis of all DEGs was based on a threshold of an adjusted *P* value < 0.05. Bar and point plots were generated by R software.

### 2.4. Protein-Protein Interaction (PPI) Network Analysis

Search Tool for the Retrieval of Interacting Genes/Proteins (STRING) (https://string-db.org/) is a website that predicts interactions among genes and proteins of interest. In the study, DEG symbols were provided as a list. The species “*Homo sapiens*” was chosen, and the minimum required interaction score was set to high confidence (0.700). A file containing protein interactions was automatically given and subsequently downloaded. Cytoscape, software for visualization of gene and protein networks, was utilized to construct the PPI network. MCODE, a plugin of Cytoscape, was used to identify potential hub genes.

### 2.5. Gene Set Enrichment Analysis (GSEA)

GSEA is a computing method for exploring the statistical significance and concordant differences of defined gene sets or pathways between two biological states. In the present study, GSEA was used to deeply analyze biological information, enlightening our understanding of relevant biological events. Two files of genomic expression data and contrast information were inputted, and the analysis was carried out using GSEA 4.0.3 software.

### 2.6. Validation of Hub Gene Prognostic Value

Further validation and survival analysis of identified hub genes were performed by the Gene Expression Profiling Interactive Analysis (GEPIA) database, which is a newly developed interactive web server for exploring RNA sequencing data from the TCGA and GTEx projects [18]. According to the overall survival (OS) and disease-free survival (DFS) data offered through GEPIA, figures displaying the influence of hub genes on PC survival were drawn. A *P* value < 0.05 was chosen as the cutoff.

## 3. Results

### 3.1. Identification of DEGs

**Figure 1:**
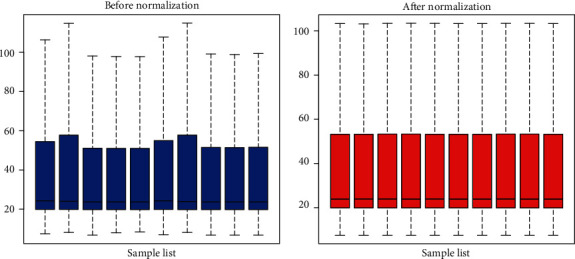
Normalization of gene expression data in samples. The blue bars represent the data before normalization, and the red bars represent the data after normalization.

Cell samples in GSE104935 were divided into two groups with 5 samples of ENZ-R and ENZ-S cells each. As exhibited in Figure 1, all expression data in each sample were normalized.

**Figure 2:**
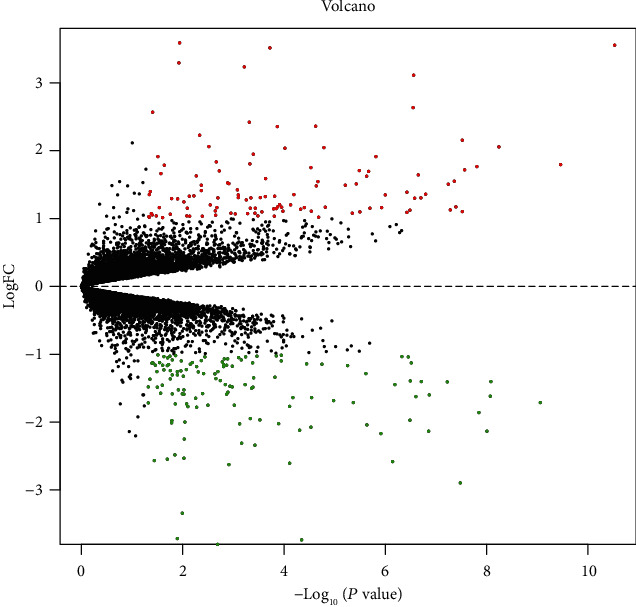
Volcano plot of DEGs between ENZ-R and ENZ-S PC cells. Red points represent upregulated genes, and green points represent downregulated genes. Genes without any significant difference are in black. The cutoffs for significant differences were ∣logFC | >2 and *P* < 0.05.

**Figure 3:**
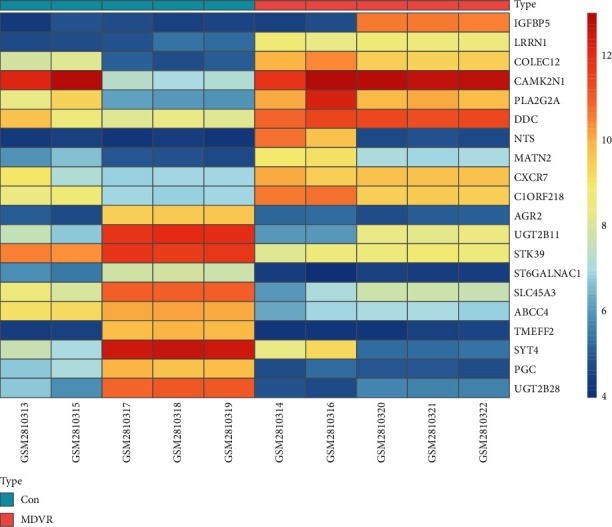
Heat map of the top 20 DEGs. Upregulated genes are marked in red, while downregulated genes are marked in blue. MDVR: ENZ-R samples; Con: ENZ-S samples.

A total of 201 DEGs containing 93 highly expressed genes and 108 genes that were expressed at a low level were identified (Table 1) according to the FC value. Volcano (Figure 2) and heat map plots (Figure 3) were constructed to show the distribution and expression of all DEGs. The top 10 upregulated DEGs were IGFBP5, LRRN1, COLEC12, CAMK2N1, PLA2G2A, DDC, NTS, MATN2, CXCR7, and C1ORF218, while the top 10 downregulated DEGs were UGT2B28, PGC, SYT4, TMEFF2, ABCC4, SLC45A3, ST6GALNAC1, STK39, UGT2B11, and AGR2.

### 3.2. GO Enrichment Analysis

**Figure 4:**
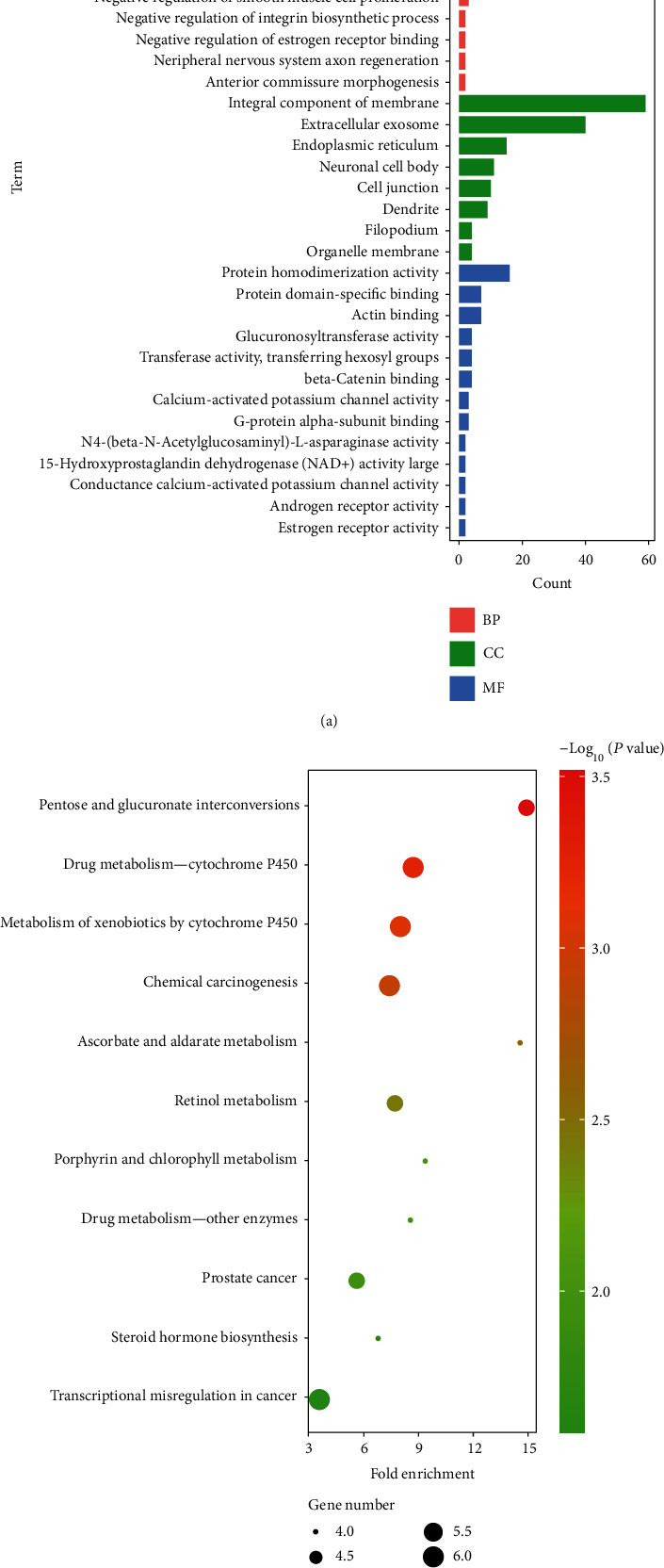
GO enrichment and KEGG pathway analyses between ENZ-S and ENZ-R cells. (a) GO enrichment analysis. DEGs were divided into 3 groups: biological processes (red bars), cell components (green bars), and molecular functions (blue bars). (b) KEGG pathway analysis. The size of the point represents the gene count, while the color gradient represents the *P* value.

As shown in Figure 4(a), three categories were included in the GO analysis: biological process (BP), molecular function (MF), and cellular component (CC). The BP terms in which the DEGs were mainly enriched included positive regulation of gene expression, metabolic processes, negative regulation of cell migration, and flavonoid biosynthetic processes. The CC terms in which the DEGs were significantly enriched were integral components of the membrane, extracellular exosomes, endoplasmic reticulum, etc. The MF terms in which the DEGs were enriched were protein homodimerization activity, protein domain-specific binding, actin binding, glucuronosyltransferase activity, etc.

The subsequent KEGG pathway analysis showed that the DEGs were mainly enriched in signaling pathways related to the metabolism of drugs and biological molecules, steroid hormone biosynthesis, transcriptional misregulation in cancer, and chemical carcinogenesis (Figure 4(b)).

### 3.3. PPI Network Construction and Hub Gene Selection

**Figure 5:**
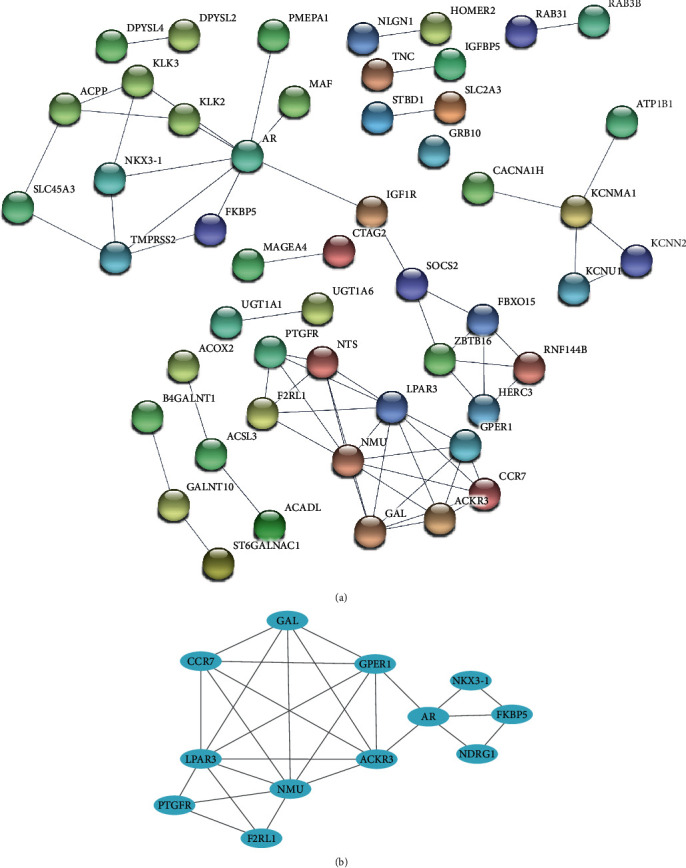
PPI network and hub genes. (a) PPI network of DEGs. (b) The identified hub genes and their interactions. Circles represent genes, and lines represent interactions among DEGs.

A total of 201 DEGs were uploaded into the STRING database. The PPI network graph (Figure 5(a)) and primary data were generated automatically. After MCODE analysis, 12 hub genes, of which 5 were upregulated and 7 were downregulated, were identified (Figure 5(b)). The upregulated hub genes were AR, ACKR3, GPER1, CCR7, and NMU, and the downregulated genes were NDRG1, FKBP5, NKX3-1, GAL, LPAR3, F2RL1, and PTGFR.

### 3.4. Identification of Potential Significant Pathways by GSEA

**Figure 6:**
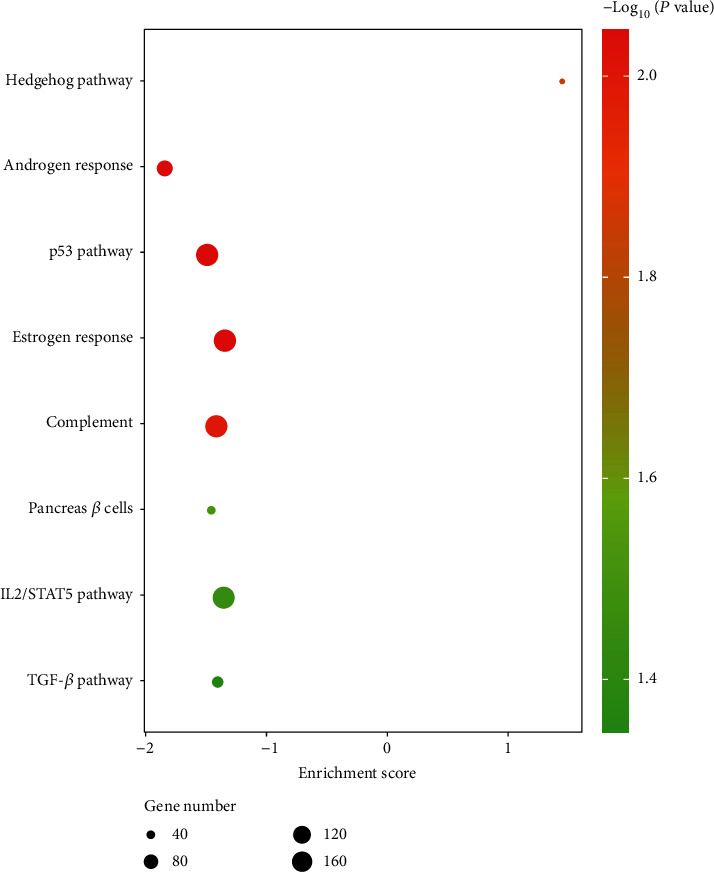
GSEA analysis of functional pathways between ENZ-S and ENZ-R PCa cells.

To further identify the potential pathways in the genesis of ENZ resistance in PCa, we performed GSEA analysis at the genomic level. In the ENZ-R cell group, the GSEA results showed that 15 gene sets were upregulated and that the hedgehog pathway was regarded as the most significant pathway (*P* < 0.05). In ENZ-S cells, 35 gene sets were downregulated, and 7 pathways were considered significantly affected, androgen response, p53, estrogen response, TNF-*α*, TGF-*β*, complement, and pancreas *β* cells (Figure 6). The seven pathways were relatively downregulated in groups of ENZ-R cells.

### 3.5. The Prognostic Value of Hub Genes in PCa Patients

**Figure 7:**
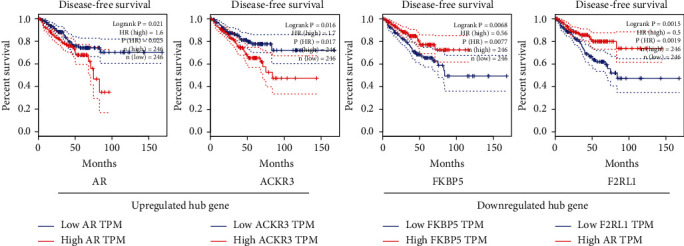
Survival analysis. Red lines represent high expression, while blue lines represent low expression.

Based on survival analysis results, we found that none of these hub genes affected the OS of PC patients. However, four hub genes were indicated to be of predictive value for DFS. The high expression of two genes (AR and ACKR3) predicted low DFS in patients with PC. In addition, patients with upregulation of another two genes (FKBP5 and F2RL1) had better DFS (Figure 7).

## 4. Discussion

ENZ resistance is a substantial barrier to PCa treatment because the occurrence of resistance for patients accepting ADT is almost universal [19]. Since the genesis of ENZ resistance involves complex biological processes including gene alterations and signal transduction, it is difficult to completely illuminate all mechanisms. Although RNA-seq analysis has been performed in the works of Zhang et al. [15] and Hwang et al. [16], their results were regarded as supplementary parts of whole experimental studies, and significant DEGs were not mentioned given the specific purpose of each study. Therefore, bioinformatics analysis of large-scale bioinformatics data at the genomic level is urgently required, with the purpose of providing potential research objectives or directions.

The aim of our study was to identify key genes and pathways at the genomic level using bioinformatics analysis. In this work, we identified a total of 201 DEGs (93 upregulated and 108 downregulated). GO enrichment analysis showed that the DEGs were enriched in the regulation of cell proliferation and migration-related gene expression, and KEGG pathway analysis found that the DEGs were closely associated with the metabolism of drugs and biological molecules, steroid hormone biosynthesis, transcriptional misregulation in cancer, and chemical carcinogenesis. After PPI network and hub gene analysis, 12 hub genes were identified, including 5 upregulated genes and 7 downregulated genes. Among the identified hub genes, some have been proven to be related to ENZ resistance or PCa progression, such as AR, ACKR3 (also named CXCR7) [20, 21], CCR7 [22], and NDRG1 [23]. In addition, NK3 homeobox1 (NKX3-1) is a prostate tumor suppressor that is associated with the DNA repair response and binds to the androgen receptor [24]. FK506 binding protein 5 (FKBP5) is a target gene of the AR downstream cascade and has been well documented in previous works [25]. The present analysis indicated that FKBP5 was downregulated, but AR was highly expressed in ENZ-resistant cells, which seemed contradictory. This result is likely a result of FKBP5 being negatively regulated by other genes in the development of ENZ resistance. Qin et al. demonstrated that nuclear receptor coactivator 2 (NCoA2 or SRC-2), a gene that promotes PCa metastasis and CRPC development, could negatively and directly regulate the FKBP5 gene at the transcriptional level in an AR-independent manner [26].

The other 5 genes (NMU, GAL, LPAR3, F2RL1, and PTGFR) are potential novel genes of ENZ resistance in PCa.

Neuromedin U (NMU) is a neuropeptide that belongs to the neuromedin family and has been shown to be related to many important activities in the nervous system [27]. A recent study showed that NMU was a novel prognostic marker of many cancer types [28]. Li et al. found that NMU was highly expressed and related to poor clinical outcomes in hepatocellular carcinoma [29]. Overexpression of NMU enhanced drug resistance in breast and lung cancer cells, whereas NMU silencing sensitized resistant cells [30].

Coagulation factor II (thrombin) receptor-like 1 (F2RL1) is known as a protease-activated receptor that is abundant in neurons, where it functions in pain, inflammation, and release of neurotransmitters [31]. Previous works have indicated that protease-activated receptor 2 (PAR2, encoded by F2RL1) functions in the regulation of carcinogenesis in diverse cancers. In hepatocellular carcinoma, PAR2 was reported to promote cell proliferation and distant metastasis by inducing EMT and to predict poor clinical outcome.

Prostaglandin F2 alpha receptor (PTGFR), a membrane receptor for prostaglandin F2 alpha, has been reported to be related to tumorigenesis and progression in endometrial adenocarcinoma. Overexpression of PTGFR was found to be a novel marker in endometrial adenocarcinoma and renal cell carcinoma [32, 33]. In prostate cancer, the next-generation sequencing analysis performed by Alkhateeb et al. identified PTGFR as a potential biomarker to predict progression [34]. Romanuik et al. performed a long serial analysis of gene expression (LongSAGE) libraries and confirmed PTGFR as a key gene that was associated with cell proliferation and in vivo progression.

Galanin (GAL) is a reported neuropeptide secreted by sensory neurons located in the gastrointestinal system [35]. GAL may play a dual role in cancer regulation because it exhibits different expression profiles in cancers. The GAL mRNA level was observed to be elevated and was significantly associated with tumor stages in colon cancer [36], while it was also indicated as a tumor suppressor correlated with low disease-free survival in head and neck squamous cell carcinoma [37].

Lysophosphatidic acid receptor 3 (LPAR3) is one of the G protein-coupled receptors that is specifically triggered by lysophosphatidic acid and is related to proliferation and aggressiveness in certain cancers [38]. Increased expression of LPAR3 was proven to increase malignancy in breast and ovarian cancers [39, 40]. LPAR3 was reported as a part of the ZEB1-AS1/miR-133a-3p/LPAR3/EGFR axis that promotes thyroid cancer progression by regulating PI3K/Akt/mTOR signaling [41]. Recent bioinformatics analysis has shown that LPAR3 is one of the hub genes in high-grade prostate cancer.

Although the above 5 hub genes are closely related to cancer regulation, their detailed functions in ENZ resistance in PCa remain unclear, and more experiments are needed in future research.

According to the GSEA results, hedgehog (HH) signaling was considered a key pathway functioning in ENZ-R cells. Research conducted by Cai et al. indicated that overexpression of the m6A methyltransferase METTL3 could upregulate GLI1, one of the main transcription factors in HH, and could enhance PCa proliferation [42]. In addition, HH has been validated as a novel therapeutic target for PC. Yang et al. designed a novel microtubule destabilizer (QW-296) that was combined with MDB5, a newly synthesized HH inhibitor, to treat taxane-resistant PC cells (PC3-TXR and DU145-TXR), and the combination achieved better anticancer efficacy than single-drug administration [43]. Sun found that an HH blocker (GANT61) and PLC knockdown synergized to impair the cell growth and colony forming ability of PCa cells and augmented sensitivity to ENZ. The above discoveries provide evidence in support of our GSEA outcome that HH signaling might be a potential key pathway in ENZ-R development. More efforts should be conducted to research the detained interaction between HH and ENZ resistance.

Previous research indicated that there was an interaction between the estrogen response and ENZ. Abazid et al. found that ENZ treatment could decrease the expression level of the estrogen receptor in PCa, and the estrogen receptor increased ENZ sensitivity to AR (+) triple-negative breast cancer [44, 45]. A study by Maughan et al. indicated that p53 inactivation was correlated with a poor response to antiandrogen drugs in CRPC [46], which is consistent with our result that the P53 pathway is downregulated in ENZ-R cells.

There are some inherent limitations in our work, resembling those of similar studies. First, we only acquired and analyzed one data matrix, GSE104935, for the analysis, since the other candidate gene sets (GSE64143 and GSE120005) contain expression data for nonduplicate samples. In the future, more genomic sequencing data for ENZ-R PC cells are urgently needed. Second, the results of our present bioinformatics analysis are based on RNA-Seq data from cell lines but not from clinical samples, which may weaken the persuasiveness of our conclusion. We will design further verification experiments on clinical PCa tissues in the future research. Third, molecular biology experiments for verifying the function of the identified hub gens and pathway are not performed, and they will be addressed as the main goal in our subsequent studies.

## 5. Conclusion

In conclusion, we identified 201 DEGs, 12 hub genes, and 8 pathways by integrated bioinformatics analysis. We speculate that these candidate genes or pathways are likely to play different roles in the generation and development of ENZ resistance. These key genes and pathways deserve more exploration and validation in future works.

## Figures and Tables

**Table 1:** Top 100 DEGs identified in GSE104935.

Expression level	DEGs (ranked in descending order of fold change)
Upregulated genes	
	IGFBP5, LRRN1, COLEC12, CAMK2N1, PLA2G2A, DDC, NTS, MATN2, ACKR3, C1ORF218, NLGN1, AUTS2, DOCK10, ATP1B1, C4ORF18, ACPP, IL1RN, LEF1, C1ORF53, MAST4, ABHD7, TMEM140, AIDA, TSPAN7, SMA4, CALD1, BASP1, GUSBL1, AR, HOXA11AS, GPER1, KCNU1, GRB10, SMA5, NUP93, HS3ST1, GPR177, BRDT, UPK1A, TSPAN8, PRR16, B4GALNT1, SERPINI1, VAV3, SLC22A3, RNF144B, NMU, TRIB1, FBXO15, SORL1, TNC, ZFHX4, RDH12, NETO1, C1ORF115, TMEM144, TMEM116, WDR91, CXORF57, FAM177B, AGA, PTPRK, DPYSL2, LUZP2, FAM113B, UGT1A6, STBD1, REEP1, SUSD4, CCR7, NF629, IRX5, INSIG2, TFAP2A, RGS17, MYRIP, C5ORF30, CCNE2, LRRC6, ENC1, C5ORF53, LAPTM4B, SLC39A8, GALNT10, NRSN2, GPR137B, LYPD6B, C16ORF45, BARD1, GSTA4, COL5A2, LGALS8, CCDC83
Downregulated genes	
	UGT2B28, PGC, SYT4, TMEFF2, ABCC4, SLC45A3, ST6GALNAC1, STK39, UGT2B11, AGR2, ADAM7, LPAR3, EDG7, ELL2, TUBA3D, PRAGMIN, TMPRSS2, ALDH1A3, IQGAP2, CTAG2, KCNN2, UGT1A1, FAM65B, ZNF533, TUBA3E, HOMER2, PMEPA1, KLK2, NKX3-1, HES6, ZBTB16, KLK3, CRYAB, HPGD, RGS2, TNFRSF19, NFIB, GLIPR2, ACSL3, CENPN, MICAL1, SPRYD5, ASRGL1, MAF, TRPM8, EAF2, CXADR, TM4SF1, FKBP5, MIPEP, LXN, SOCS2, ACOX2, HERC3, MGC18216, RALYL, ELOVL5, PDE9A, DKFZP761P0423, OSBPL8, UAP1, FAM105A, KCNMA1, PRUNE2, AFF3, MAPK6, KRTAP13-2, RAB31, KIAA0367, DPYSL4, AZGP1, MAP1LC3A, WDR72, DSC2, ZNF812, APOD, VIM, FHDC1, SLC2A3, MMP12, KRT19, SORD, MYCBP2, F2RL1, DPP4, TMEM45B, RAB3B, ZNF385B, KCNK1, NDRG1, ABHD2, BICD2, PTGFR, GPR126, SLC10A7, TSKU, GAL, ANKRD37, FLJ22795, MAGEA4, ACADL, ARHGAP28, CACNA1H, C19ORF48, FAM174B, PTGR1, FGFR3, NMD3

## Data Availability

The data of GSE104935 used in our study are available via GEO (https://www.ncbi.nlm.nih.gov/geo/) or by appropriate request to the authors.

## References

[1] Siegel R. L., Miller K. D., Jemal A. (2018). Cancer statistics, 2019. *CA: a Cancer Journal for Clinicians*.

[2] Crawford E. D., Schellhammer P. F., McLeod D. G. (2018). Androgen receptor targeted treatments of prostate cancer: 35 years of progress with antiandrogens. *The Journal of Urology*.

[3] Tan M. H. E., Li J., Xu H. E., Melcher K., Yong E. L. (2015). Androgen receptor: structure, role in prostate cancer and drug discovery. *Acta Pharmacologica Sinica*.

[4] Davis I. D., Martin A. J., Stockler M. R. (2019). Enzalutamide with standard first-line therapy in metastatic prostate cancer. *The New England Journal of Medicine*.

[5] Scher H. I., Fizazi K., Saad F. (2012). Increased survival with enzalutamide in prostate cancer after chemotherapy. *The New England Journal of Medicine*.

[6] Scher H. I., Beer T. M., Higano C. S. (2010). Antitumour activity of MDV3100 in castration-resistant prostate cancer: a phase 1-2 study. *Lancet*.

[7] Chen X., Lu J., Xia L., Li G. (2018). Drug resistance of enzalutamide in CRPC. *Current Drug Targets*.

[8] Yamamoto Y., Loriot Y., Beraldi E. (2015). Generation 2.5 antisense oligonucleotides targeting the androgen receptor and its splice variants suppress enzalutamide-resistant prostate cancer cell growth. *Clinical Cancer Research : an official journal of the American Association for Cancer Research*.

[9] Zong Y., Goldstein A. S. (2013). Adaptation or selection--mechanisms of castration-resistant prostate cancer. *Nature Reviews Urology*.

[10] Antonarakis E. S., Lu C., Wang H. (2014). AR-V7 and resistance to enzalutamide and abiraterone in prostate cancer. *The New England Journal of Medicine*.

[11] Nadiminty N., Tummala R., Liu C. (2013). NF-*κ*B2/p52 induces resistance to enzalutamide in prostate cancer: role of androgen receptor and its variants. *Molecular Cancer Therapeutics*.

[12] Carver B. S., Chapinski C., Wongvipat J. (2011). Reciprocal feedback regulation of PI3K and androgen receptor signaling in PTEN-deficient prostate cancer. *Cancer Cell*.

[13] Isikbay M., Otto K., Kregel S. (2014). Glucocorticoid receptor activity contributes to resistance to androgen-targeted therapy in prostate cancer. *Hormones and Cancer*.

[14] Miyamoto D. T., Zheng Y., Wittner B. S. (2015). RNA-Seq of single prostate CTCs implicates noncanonical Wnt signaling in antiandrogen resistance. *Science*.

[15] Zhang Z., Cheng L., Li J. (2018). Inhibition of the Wnt/*β*-catenin pathway overcomes resistance to enzalutamide in castration-resistant prostate cancer. *Cancer Research*.

[16] Hwang J. H., Seo J.-H., Beshiri M. L. (2019). CREB5 promotes resistance to androgen-receptor antagonists and androgen deprivation in prostate cancer. *Cell Reports*.

[17] Huang D. W., Sherman B. T., Lempicki R. A. (2009). Systematic and integrative analysis of large gene lists using DAVID bioinformatics resources. *Nature Protocols*.

[18] Tang Z., Li C., Kang B., Gao G., Li C., Zhang Z. (2017). GEPIA: a web server for cancer and normal gene expression profiling and interactive analyses. *Nucleic Acids Research*.

[19] Chen W. S., Aggarwal R., Zhang L. (2019). Genomic drivers of poor prognosis and enzalutamide resistance in metastatic castration-resistant prostate cancer. *European Urology*.

[20] Li S., Fong K.-W., Gritsina G. (2019). Activation of MAPK signaling by CXCR7 leads to enzalutamide resistance in prostate cancer. *Cancer Research*.

[21] Snow O., Lallous N., Singh K., Lack N., Rennie P., Cherkasov A. (2019). Androgen receptor plasticity and its implications for prostate cancer therapy. *Cancer Treatment Reviews*.

[22] Du R., Tang G., Tang Z., Kuang Y. (2018). Ectopic expression of CC chemokine receptor 7 promotes prostate cancer cells metastasis via Notch1 signaling. *Journal of Cellular Biochemistry*.

[23] Sharma A., Mendonca J., Ying J. (2017). The prostate metastasis suppressor gene NDRG1 differentially regulates cell motility and invasion. *Molecular Oncology*.

[24] Bowen C., Zheng T., Gelmann E. P. (2015). NKX3.1 suppresses TMPRSS2-ERG gene rearrangement and mediates repair of androgen receptor-induced DNA damage. *Cancer Research*.

[25] Song I.-S., Jeong Y. J., Kim J. (2020). Pharmacological inhibition of androgen receptor expression induces cell death in prostate cancer cells. *Cellular and Molecular Life Sciences*.

[26] Qin J., Lee H. J., Wu S. P. (2014). Androgen deprivation-induced NCoA2 promotes metastatic and castration-resistant prostate cancer. *The Journal of Clinical Investigation*.

[27] De Prins A., Van Eeckhaut A., Smolders I., Tourwé D., Ballet S. (2019). Neuromedin U and structural analogs: an overview of their Structure, Function and Selectivity. *Current Medicinal Chemistry*.

[28] Przygodzka P., Soboska K., Sochacka E., Boncela J. (2019). Neuromedin U: a small peptide in the big world of cancer. *Cancers*.

[29] Li Q., Han L., Ruan S. (2020). The prognostic value of neuromedin U in patients with hepatocellular carcinoma. *BMC Cancer*.

[30] Wu Y., McRoberts K., Berr S. S., Frierson H. F., Conaway M., Theodorescu D. (2007). Neuromedin U is regulated by the metastasis suppressor RhoGDI2 and is a novel promoter of tumor formation, lung metastasis and cancer cachexia. *Oncogene*.

[31] Saito T., Bunnett N. W. (2005). Protease-activated receptors: regulation of neuronal function. *Neuromolecular Medicine*.

[32] Akiyama K., Ohga N., Maishi N. (2013). The F-prostaglandin receptor is a novel marker for tumor endothelial cells in renal cell carcinoma. *Pathology International*.

[33] Sales K. J., Milne S. A., Williams A. R. W., Anderson R. A., Jabbour H. N. (2004). Expression, localization, and signaling of prostaglandin F2 alpha receptor in human endometrial adenocarcinoma: regulation of proliferation by activation of the epidermal growth factor receptor and mitogen-activated protein kinase signaling pathways. *The Journal of Clinical Endocrinology and Metabolism*.

[34] Alkhateeb A., Rezaeian I., Singireddy S., Cavallo-Medved D., Porter L. A., Rueda L. (2019). Transcriptomics signature from next-generation sequencing data reveals new transcriptomic biomarkers related to prostate cancer. *Cancer Informatics*.

[35] Petrescu A. D., Grant S., Williams E. (2020). Coordinated targeting of galanin receptors on cholangiocytes and hepatic stellate cells ameliorates liver fibrosis in multidrug resistance protein 2 knockout mice. *The American Journal of Pathology*.

[36] Kim K. Y., Kee M. K., Chong S. A., Nam M. J. (2007). Galanin is up-regulated in colon adenocarcinoma. *Cancer Epidemiology, Biomarkers & Prevention : a publication of the American Association for Cancer Research, cosponsored by the American Society of Preventive Oncology*.

[37] Misawa K., Kanazawa T., Misawa Y. (2013). Galanin has tumor suppressor activity and is frequently inactivated by aberrant promoter methylation in head and neck cancer. *Translational Oncology*.

[38] Im D. S., Heise C. E., Harding M. A. (2000). Molecular cloning and characterization of a lysophosphatidic acid receptor, Edg-7, expressed in prostate. *Molecular Pharmacology*.

[39] Yu S., Murph M. M., Lu Y. (2008). Lysophosphatidic acid receptors determine tumorigenicity and aggressiveness of ovarian cancer cells. *Journal of the National Cancer Institute*.

[40] Liu S., Murph M., Panupinthu N., Mills G. B. (2014). ATX-LPA receptor axis in inflammation and cancer. *Cell Cycle*.

[41] Xia W., Jie W. (2020). ZEB1-AS1/miR-133a-3p/LPAR3/EGFR axis promotes the progression of thyroid cancer by regulating PI3K/AKT/mTOR pathway. *Cancer Cell International*.

[42] Cai J., Yang F., Zhan H. (2019). RNA m6A methyltransferase METTL3 promotes the growth of prostate cancer by regulating hedgehog pathway. *OncoTargets and Therapy*.

[43] Yang R., Chen H., Guo D. (2019). Polymeric micellar delivery of novel microtubule destabilizer and hedgehog signaling inhibitor for treating chemoresistant prostate cancer. *The Journal of Pharmacology and Experimental Therapeutics*.

[44] Anestis A., Sarantis P., Theocharis S. (2019). Estrogen receptor beta increases sensitivity to enzalutamide in androgen receptor-positive triple-negative breast cancer. *Journal of Cancer Research and Clinical Oncology*.

[45] Abazid A., Martin B., Choinowski A. (2019). The androgen receptor antagonist enzalutamide induces apoptosis, dysregulates the heat shock protein system, and diminishes the androgen receptor and estrogen receptor *β*1 expression in prostate cancer cells. *Journal of Cellular Biochemistry*.

[46] Maughan B. L., Guedes L. B., Boucher K. (2018). p53 status in the primary tumor predicts efficacy of subsequent abiraterone and enzalutamide in castration-resistant prostate cancer. *Prostate Cancer and Prostatic Diseases*.

